# Autonomous Trajectory Generation Comparison for De-Orbiting with Multiple Collision Avoidance

**DOI:** 10.3390/s22187066

**Published:** 2022-09-19

**Authors:** Karla Raigoza, Timothy Sands

**Affiliations:** Sibley School of Mechanical and Aerospace Engineering, Cornell University, Ithaca, NY 14853, USA

**Keywords:** autonomous trajectory optimization, trajectory generation, Pontryagin, space debris, collision avoidance

## Abstract

Over the past four decades, space debris has been identified as a growing hazard for near-Earth space systems. With limited access to space debris tracking databases and only recent policy advancements made to secure a sustainable space environment and mission architecture, this manuscript aims to establish an autonomous trajectory maneuver to de-orbit spacecrafts back to Earth using collision avoidance techniques for the purpose of decommissioning or re-purposing spacecrafts. To mitigate the risk of colliding with another object, the spacecraft attitude slew maneuver requires high levels of precision. Thus, the manuscript compares two autonomous trajectory generations, sinusoidal and Pontragin’s method. In order to determine the Euler angles (roll, pitch, and yaw) necessary for the spacecraft to safely maneuver around space debris, the manuscript incorporates way-point guidance as a collision avoidance approach. When the simulation compiled with both sinusoidal and Pontryagin trajectories, there were differences within the Euler angle spacecraft tracking that could be attributed to the increased fuel efficiency by over five orders of magnitude and lower computation time by over 15 min for that of Pontryagin’s trajectory compared with that of the sinusoidal trajectory. Overall, Pontryagin’s method produced an autonomous trajectory that is more optimal by conserving 37.9% more fuel and saving 40.5% more time than the sinusoidal autonomous trajectory.

## 1. Introduction

Since the 20th century Space Race, spacecraft have been launched into near-Earth orbits, with the majority of them and their successors remaining in space, even after their operational lifetime. Having these retired spacecrafts accumulate in Earth orbits as newer spacecraft are deployed creates hazards, termed space debris, the long-term effects of which have been explored. As early as 1978, NASA scientists Donald Kessler and Burton Cour-Palais mathematically predicted that with enough spacecraft mass orbiting Earth, they will begin to collide with each other at an exponential rate to produce more and smaller objects in space, further contributing to precarious space debris. With an increase in satellites in low-Earth orbit (LEO), they concluded that this cascading flux of spacecraft collisions will create an orbital debris belt around Earth limiting the ability to successfully send new spacecraft into and beyond Earth’s orbit [1]. Over four decades later, the amount of LEO satellite deployments has increased, as foreseen by Kessler and Cour-Palais, and there is a growing concern that the theoretical spacecraft critical mass needed for the exponential space debris collision phenomena that is the Kessler Syndrome might become a reality.

While space missions and launches have not been modified to mitigate the Kessler Syndrome directly, there still remains a call for a more sustainable space environment to either remove current space debris or design a new space mission architecture to include the return/removal of spacecrafts in-orbit in place of decommissioning. Most recently, in April 2022, the United States government outlined the first policy document by any government to combat space debris openly. In the In-Space Servicing, Assembly, and Manufacturing National Strategy report, the National Science and Technology Council included “timely debris collection and removal” among other demands for renewable in-space spacecraft design policies [2]. This expectation for a redesign of space mission architectures to moderate the effects of the Kessler Syndrome is the premise of this manuscript.

**Figure 1:**
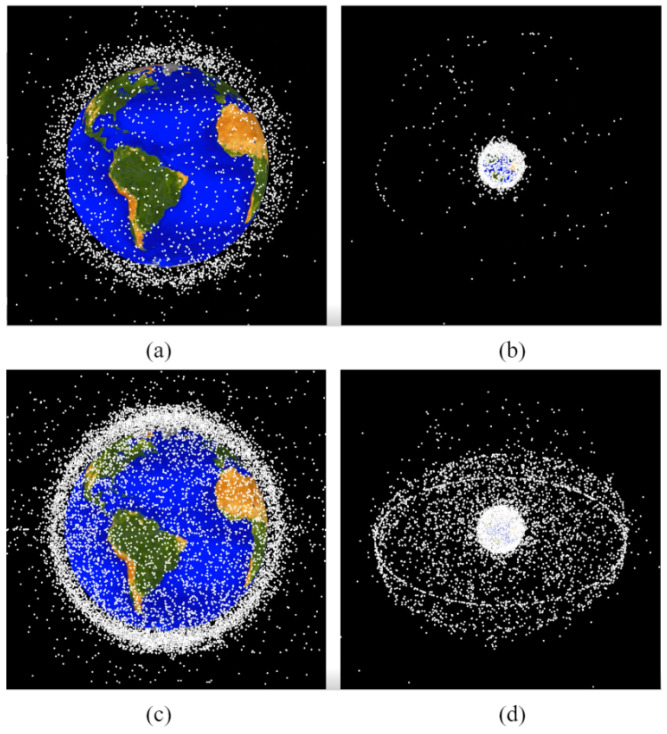
Progression of the space debris formation surrounding Earth from 1975 (**a**,**b**) to 2019 (**c**,**d**) with a minimum of 10 cm in diameter as modeled by NASA’s Orbital Debris Program Office (ODPO) [6]. Image usage is consistent with NASA policy, “NASA content (images, videos, audio, etc.) are generally not copyrighted and may be used for educational or informational purposes without needing explicit permissions.” [7].

For a spacecraft to be externally removed or internally maneuvered from its current position, precision is necessary so not to collide with any other spacecrafts and form more space debris. An object traveling at 10 km/s with a mass of 1 kg has the potential to split a 1000 kg spacecraft into objects of at least 1 kg if it collides with a high-density component [3]. To alleviate this risk, corporations such as the North American Aerospace Defense Command (NORAD), Lockheed Martin, and the National Aeronautics and Space Administration (NASA) collected data of launched spacecrafts. The NORAD was the first to develop a database, titled CelesTrak, of launch dates, heat shields, and booster rockets for spacecrafts in the late 1950s [4]. In 2020, Lockheed Martin partnered with the United States Space Force (USSF)-assembled Space Fence, which has the power to detect, monitor, track, and characterize space debris within LEO altitudes. Today, Space Fence is the most advanced database of space debris that is revolutionizing the way the near-Earth space environment is modeled [5]. Additionally, NASA has also been developing its own 3D database of Earth’s orbiting space debris within its Orbital Debris Program Office (ODPO) [6]. To better envision the progression of orbital space debris, the comparison of cataloged objects of at least 10 cm in diameter between 1975 and 2019 is shown below in Figure 1. Given that space debris tracking databases have been created but not made available to the public, this manuscript adopts a collision avoidance approach to generate spacecraft slew maneuvers so that the specific locations of space debris can be easily adapted to the model.

Applying autonomous trajectory generation methods whose provenance lies in [8]; this manuscript models a spacecraft as it de-orbits from LEO using a collision avoidance technique four times. Fine precision for this maneuver is required to avoid collisions with space debris objects; thus, accuracy, fuel consumption, and run time for two autonomous slew trajectories are analyzed. This comparison of autonomous attitude slew maneuvers is between the sinusoidal trajectory [8,9] and Pontryagin’s method [10], where fuel consumption is optimally minimized. When implementing a collision avoidance approach, the model will operate using way-point guidance to manually enter the positions of anticipated space debris. As the spacecraft travels back to Earth’s surface, this maneuver provides an opportunity for the spacecraft to be decommissioned by burning up in Earth’s atmosphere or to be repurposed for future space missions if it returns with its structural integrity still intact; both are in compliance with the space debris removal policy as established in the In-Space Servicing, Assembly, and Manufacturing National Strategy Report [2]. With this applicability within the aerospace industry, it is crucial to note the development of autonomous trajectories and collision avoidance measures made in academia.

### 1.1. Literature Review

The current research state of autonomous trajectory navigation [11] and of collision avoidance techniques centers around support for deterministic artificial intelligence [9], which necessitates autonomous trajectory generation [10]. Due to the nature of the cutting-edge collision avoidance methodology, there is more research completed in the field of autonomous and optimal trajectory than there is with collision avoidance. The inclusion of autonomous collision avoidance into trajectory generation for deterministic artificial intelligence seems to be an area ripe with opportunity. Exactly such is proposed in this manuscript.

In the field of aerospace, optimal techniques are enticing, as with any other field, but it is especially difficult to demonstrate and confirm without testing. As a result, there have been efforts to set position and attitude as constraints as well as disturbance restrictions [12]. Additionally, research has shown alternative ways to overcome the limited control actuators aboard spacecraft when it comes to trajectory generation [13,14]. Given that control moment gyroscopes are a common component in attitude and control, they still pose an analytical risk when considering gyroscope singularity [15]. To mitigate this computational hazard, guidance attitude trajectories are only designed for small bodies [16]. With this growing comfortability of spacecraft trajectory generation, it is apparent that there has been a more recent resurgence towards autonomous and optimal attitude generation [17]. This can be seen in the orbit assembly and agile maneuvering requirement of constellation satellites [18]. Given this literature review, autonomous spacecraft trajectory generation methods along with collision avoidance techniques were identified.

### 1.2. Problem Overview

The concepts of the Kessler Syndrome and the obligation for a more sustainable and renewable space environment for the future of space missions guide the significance of this manuscript. Along with the appropriated industry feasibility and academically reviewed research areas, the manuscript proposes the following novelties:

1. Generate sinusoidal and Pontryagin-based autonomous slew trajectories.
2. Generate way-point guidance Euler angles (roll, pitch, and yaw) as a collision avoidance technique.
3. Compare the performance and accuracy of the generated trajectories as the spacecraft de-orbits using the commanded Euler angles and the way-point guidance Euler angles.
4. Verify the results of the simulation using the normalization of the quaternions.

The remainder of the manuscript presents the methods utilized and the governing equations in generating the autonomous trajectory as well as the collision avoidance techniques for a de-orbiting spacecraft from LEO (Section 2). Furthermore, the spacecraft simulation results for both trajectories are detailed (Section 3). Lastly, there is a validation discussion of the results and a comparison of the trajectory results (Section 4).

## 2. Methods and Model

The following section consists of the autonomous trajectory and collision avoidance methods along with their respective governing equations used in modeling the spacecraft de-orbiting maneuver with three-dimensional (rotational and translational) motion and six degrees of freedom. To create this analytical simulation, the spacecraft was decomposed into its nine major subsystems in MatLab Simulink: commanded Euler angles, way-point guidance, trajectory, actuators and control, sensors and observers, disturbances, dynamic translational kinetics, and natural forces (see Appendix A for more detail).

### 2.1. Model Mechanics

**Figure 2:**
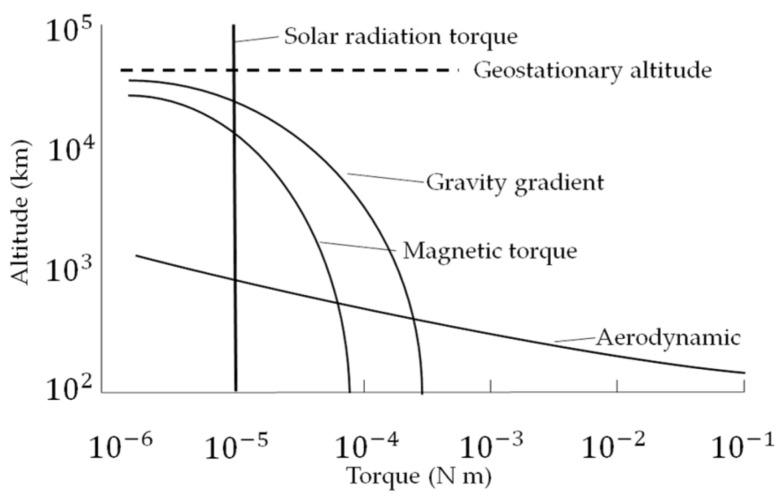
Spacecraft disturbances regarding order of magnitude approximations of external space environment torques as a function of altitude. For LEO (altitudes of <$10^{3}$ km), it is apparent that aerodynamic drag is a dominant force. Courtesy of Peter Yao.

As the simulation runs from left to right for a duration of 1000 s, the commanded Euler angles are manipulated in accordance with the controllers (designed to use a PID controller for sinusoidal trajectory generation and time optimal control for Pontryagin’s trajectory), dynamics (using quaternion kinematics along with a Direction Cosine Matrix), disturbances (including magnetic, aerodynamic, solar and gravity gradient disturbances), translational kinetics (as well as rotational), and natural forces (gravity and drag) to most accurately mimic a spacecraft trajectory in a space environment. Since the maneuver is completed in LEO, the external forces active on the spacecraft are principally aerodynamic drag (Figure 2). Accounting for the spacecraft external disturbances, the atmospheric effects of the sun were considered, as the sun’s beta angle is tracked and the atmosphere has variable density (including the effects of eclipses).

In generating the model for this simulation, a crucial equation is that of the full-coupled non-linear translational and rotational motion equation that includes the relative, Euler, coriolis and centrifugal forces (Equation (1)).

(1) $$\sum F=\underset{\mathrm{Relative}}{\underset{︸}{ma}}+\underset{\mathrm{Euler}}{\underset{︸}{m\frac{d\omega }{dt}\times r}}+\underset{\mathrm{Coriolis}}{\underset{︸}{2m\omega \times v}}+\underset{\mathrm{Centrifugal}}{\underset{︸}{m\omega \times \omega \times r}}$$

For a more detailed derivation on this equation, Sands (2022) provides a more in-depth discussion [19]. The terms of the 6 degree of freedom motion equation displayed overhead are defined in Table 1. Above, ω is coupled, so as the altitude of the spacecraft changes to avoid collisions with space debris, the orbit is controlled by conserving momentum, swapping linear for angular momentum. Therefore, it is possible to control the orbit without using fuel by solving for attitude Euler angles, which thus is the basis of the model simulation.

Given the broad scope of the Simulink model, it is easier to visualize the mechanics of how it operates in the space environment. While the simulation includes nine subsystems, not all components are analyzed in this manuscript. The subsystems that are the focal points of this manuscript are the way-point guidance and trajectory components, which will be specified in greater detail below, including the methodology and governing equations.

### 2.2. Autonomous Trajectory Generation

The trajectory subsystem consists of the autonomous trajectory generation. Since 2020, sinusoidal trajectory approaches have been used for their simplicity and applied to deterministic artificial intelligence space systems, which has created the benchmark for this manuscript [20]. Similarly, sinusoidal trajectory generation can be viewed as a traditional approach and, when juxtaposed with that of a fuel-optimized trajectory using Pontryagin’s method, it has resulted in a lower control effort [10]. To further compare the computational power of sinusoidal and Pontryagin’s optimal trajectory generation, this manuscript has incorporated collision avoidance techniques. For the purposes of this manuscript, there are two trajectories that are calculated and whose results are compared with each other: sinusoidal trajectory and Pontryagin’s optimal trajectory (as detailed in Appendix A).

#### 2.2.1. Sinusoidal Approximation Trajectory Method

One way to command a spacecraft to a trajectory autonomously is by using a sinusoidal approximation. When a spacecraft applies a new trajectory and assumes an instantaneous attitude maneuver, the trajectory would be represented as a step function. While this would be ideal in terms of time efficiency, in reality, instantaneous slew is not possible. Thus, for the purposes of creating a smooth, differentiable trajectory in this analytical model, it can be assumed that the trajectory generation scheme is made using simple harmonic motion represented as a sinusoidal piece-wise wave function [21].

In providence of this method, a derivation is best detailed in Sands (2020) [9], where the position, velocity and acceleration shown below on the left side of Equations (2)–(4), respectively, are the generalized autonomous trajectory equations. When applied to the desired 30° yaw maneuver, the resulting simplified position, velocity, and acceleration trajectory equations are depicted on the right side of Equations (2)–(4), respectively. The terms of the sinusoidal trajectory equations stated below are defined in Table 2.

(2) $$z_{d}=A_{0}+\frac{\left(A-A_{0}\right)}{2}\left[1+sin\left(\frac{\pi }{\Delta t_{maneuver}}\right)\left(t+\frac{3\Delta t_{maneuver}}{2}\right)-\Delta t_{quiescant}\right]⟶\frac{1}{2}\left[sin\left(\frac{\pi }{t_{maneuver}}\left(t+2.5\right)\right)+1\right]$$

(3) $$\dot{z_{d}}=\frac{\left(A-A_{0}\right)}{2}\left(\frac{\pi }{\Delta t_{maneuver}}\right)cos\left(\frac{\pi }{\Delta t_{maneuver}}\left(t+\frac{3\Delta t_{maneuver}}{2}\right)-\Delta t_{quiescant}\right)⟶\frac{30}{77.1239}\left(\frac{1}{2}\right)\frac{\pi }{t_{maneuver}}\left[cos\left(\frac{\pi }{t_{maneuver}}\left(t+2.5\right)\right)+1\right]$$

(4) $$\ddot{z_{d}}=-\frac{\left(A-A_{0}\right)}{2}{\left(\frac{\pi }{\Delta t_{maneuver}}\right)}^{2}sin\left(\frac{\pi }{\Delta t_{maneuver}}\left(t+\frac{3\Delta t_{maneuver}}{2}\right)-\Delta t_{quiescant}\right)⟶\frac{-30}{77.1239}\left(\frac{1}{2}\right)\frac{\pi }{t_{maneuver}^{2}}sin\left(\frac{\pi }{t_{maneuver}}\left(t+2.5\right)\right)$$

To analyze the sinusoidal trajectory results, it is noted that the desired Euler angle appears in the amplitude of the sine wave. When Equations (2)–(4) are multiplied with the desired Euler angles (roll, pitch, and yaw), it produces the commanded Euler angles in terms of the angle, rate, and angular rate trajectory, respectively.

#### 2.2.2. Pontryagin’s Method Optimal Trajectory

Another approach in generating an autonomous trajectory for a spacecraft is by utilizing Pontryagin’s minimum principle as an optimization tool. While the sinusoidal trajectory applies a sinusoidal approximation of the trajectory, Pontryagin’s minimum principle presents an alternate approach through its formation of a boundary value problem. By deriving and incorporating Pontryagin’s minimum principle, an optimal trajectory is determined with respect to the cost function (for the purposes of this manuscript it is fuel consumption). The anticipated results of Pontryagin’s method are to be faster and consume less fuel than that of the sinusoidal trajectory.

In applying Pontryagin’s method to this simulation, the prominent motion of the spacecraft is that of the rotation. As derived from Newton’s second law and Euler’s equations, the governing equation for the rotational motion is represented in Equation (5), assuming the spacecraft is a rigid body [20].

(5) $$\sum \tau =I\ddot{\theta }+\omega \times I\omega$$

where *I* is the mass moment of inertia, $\ddot{\omega }$ is the angular acceleration, and ω is the angular velocity of the spacecraft. The first term is characterized as the double integrator and the second term can be recognized as the transport theorem. Together, the sum of the double integrator and transport theorem (which are in compact vector–matrix notation) produce the summation of the major external torques. In this equation, the double integrator is the dominant term, which is where Pontryagin’s method can be applied. To generate an autonomous optimal trajectory operated by Pontryagin’s minimum principle, the equation of focus is shown below (Equation (6)) along with its conditions. The cost function is represented in the integral as the force exerted from the spacecraft, which is assumed to be proportional to the fuel consumption. The resulting force is applicable to the three-dimensional rotational motion torque. With Pontryagin’s method, it approximates the external torque required for rotational and translational motion. The terms of Pontryagin’s trajectory equations declared underneath are defined in Table 3.

(6) $$Minimize:J\left[x,u\right]=\frac{1}{2}\int_{t_{0}}^{t_{f}}u^{T}u\;dt$$

$$\begin{array}{c} \mathrm{Subject}\;\mathrm{to}:\;\dot{x}=v\\ \dot{v}=f/\left[M\right]\\ \left(x_{0},v_{0}\right)=\left(0,0\right)\\ \left(x_{1},v_{1}\right)=\left(-1,0\right)\\ t_{0}=0\\ t_{f}=1 \end{array}$$

As a heuristic form of optimization, Pontryagin’s method proceeds the derivation of the Hamiltonian from Equation (6) and is used to form the boundary value problem with the addition of $f=\frac{-\lambda_{v}}{\left[M\right]}$, $\lambda_{x}=a$, and $\lambda_{v}=at+b$ to the state conditions, where $\lambda_{x}$ and $\lambda_{v}$ are the Lagrangian coefficients for the position and velocity, respectively, *a* and *b* are constants, and *m* is the mass of the spacecraft. Incorporating these new definitions of state conditions, the force, velocity, and position equations can be reformulated in terms of constants a,b,c,d, and *m*. Solving at the initial and final conditions produces values for the constants that express the optimal force, rate, and state outputs with respect to time, displayed in Equations (7)–(9).

(7) $$f^{*}=12t-6$$

(8) $$v^{*}=\frac{6t^{2}-6t}{\left[M\right]}$$

(9) $$x^{*}=\frac{2t^{3}-3t^{2}}{\left[M\right]}$$

#### 2.2.3. Scaling and Balancing

To assure the accuracy of very small and large numbers in the computational mathematical analysis, the mass was scaled to unity (Equation (10)), where the real mass is divided by a number very similar to that of the real mass that yields a mass term approximation of 1 (i.e., unity). By scaling equations with universally known values, it allows developments and observations to be widely adapted to state spaces that initially it was not designed for.

(10) $${\left[M\right]}_{unitized}=\frac{{\left[M\right]}_{real}}{{\left[M\right]}_{nominal}}$$

Now that the optimal force, rate, and state equations are calculated, a balancing of equations is needed to unscale and obtain equations with the original mass. In multiplying the nominal mass to Equations (7)–(9), it provides the fully scaled and balanced optimal force, rate, and state Equations (11)–(13).

(11) $$f^{*}=\left[M\right]\left(12t-6\right)$$

(12) $$v^{*}=6t^{2}-6t$$

(13) $$x^{*}=2t^{3}-3t^{2}$$

When the Pontryagin’s trajectory equations are multiplied with the desired Euler angles (roll, pitch, and yaw), it produces the commanded Euler angles in terms of angle, rate, and the angular rate trajectory, respectively. Since Pontryagin’s method is conducted with respect to the principal frame, the commanded Euler angles are converted to the body frame before being transferred to the actuators and control subsystem.

### 2.3. Collision Avoidance

The other critical subsystem for this manuscript is that of way-point guidance that consists of the derivation of the commanded Euler angles necessary for collision avoidance. As mentioned in the literature review, collision avoidance is a topic that is still being formulated in the research, thus there is less of a precedented method to approach this simulation. For the purposes of this manuscript, the collision avoidance technique used is that of way-point guidance, which acts as an estimation for determining necessary Euler angles for a maneuver. The following section presents the methodology behind way-point guidance to generate the required commanded Euler angles for the anticipated space debris that coincides within the trajectory.

#### Way-Point Guidance Method

The way-point guidance technique operates on the assumption that the location of the space debris coinciding within the projected trajectory is known. This method of collision avoidance is constructed by manually setting the duration of slew time as well as the miss distance, which is the distance tolerance between both objects when they are passing each other, and the forward travel distance, which is the distance between the spacecraft and the space debris as the spacecraft is traveling forward. These parameters are related to each other in Equation (14) below.

(14) $$atan=\frac{Miss\;Distance}{Forward\;Travel\;Distance}$$

For the purposes of this manuscript, it is assumed that there are four space debris objects whose positions in relation to the planned trajectory are known. Each set of collision avoidance commanded Euler angles are then propagated throughout the rotational and translational model of the spacecraft motion to produce a trajectory.

## 3. Results

With the description of the methods used to establish the model, the corresponding simulation results are detailed in this section. The proposed simulation was run with a spacecraft mass of 100 kg de-orbiting at a starting altitude of 1000 km for a total of 600 s with four way-point guidance maneuvers ($t_{1}$ = 50 s, $t_{2}$ = 150 s, $t_{3}$ = 300 s, and $t_{4}$ = 450 s), all with a slew time of 100 s and a time step of 0.001 s. Each way-point guidance maneuver was set to the same yaw degree change with a miss distance of 0.3 km and a forward travel distance of 10,000 km. The initial commanded Euler angle was set for a 30-degree yaw maneuver and was run using the fourth-order Runge Kutta solver.

### 3.1. Sinusoidal Autonomous Trajectory

**Figure 3:**
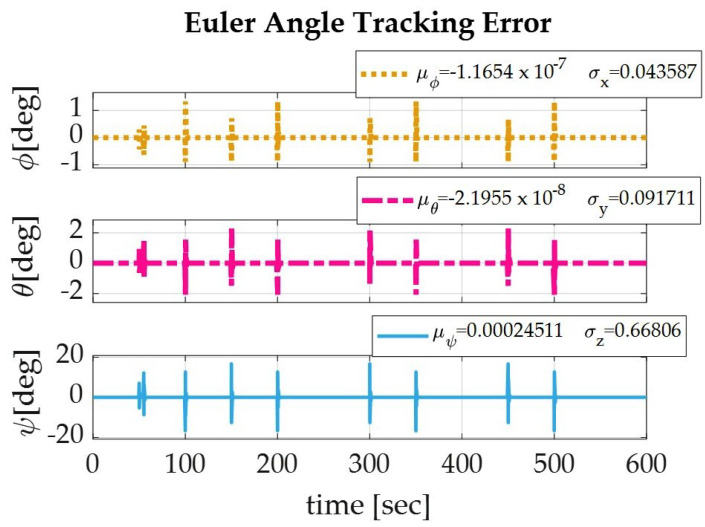
The Euler angle tracking errors for a spacecraft de-orbiting from LEO altitude (1000 km) using a sinusoidal autonomous trajectory for roll (yellow), pitch (pink), and yaw (blue).

**Figure 4:**
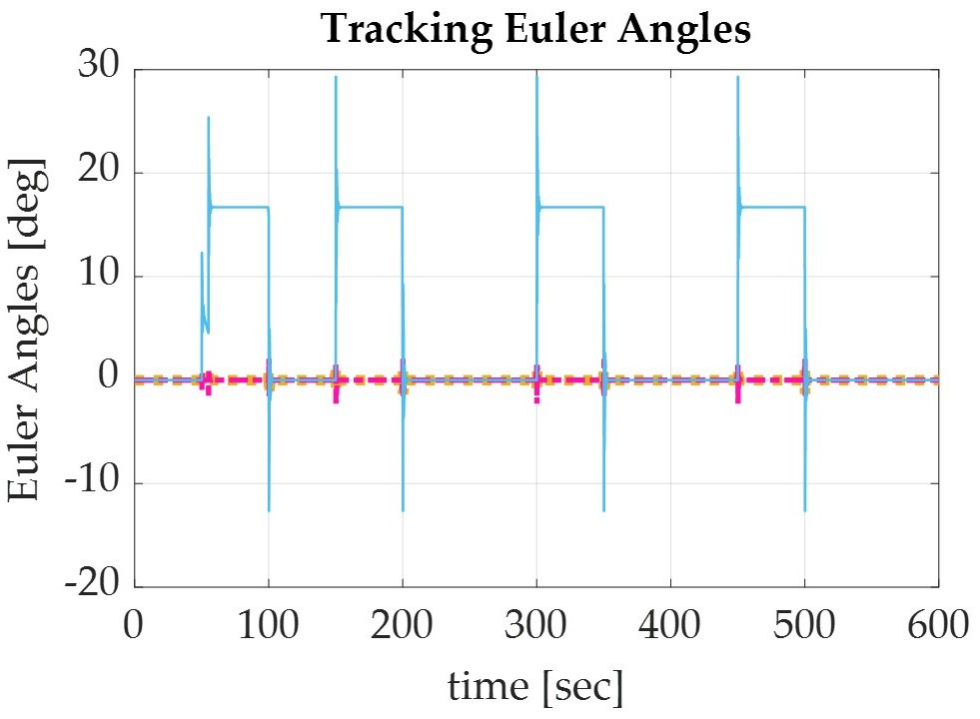
Graph of the Euler angle tracking for a spacecraft de-orbiting from LEO altitude (1000 km) using a sinusoidal autonomous trajectory for roll (yellow), pitch (pink), and yaw (blue).

**Figure 5:**
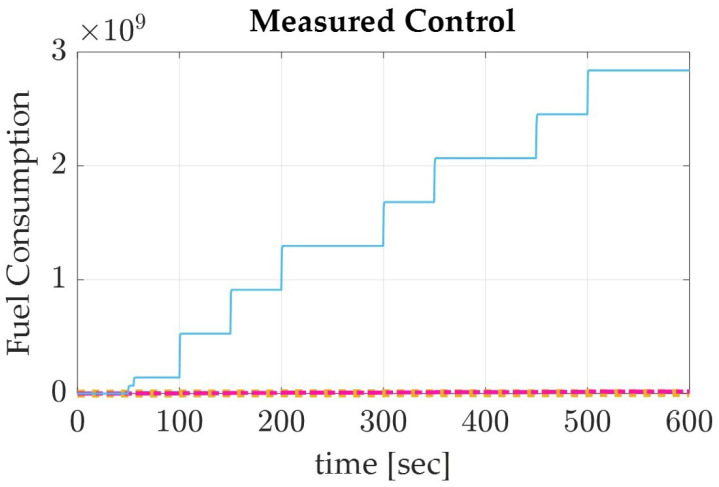
The control measured in terms of thrust applied, which can be estimated for fuel consumption, for a de-orbiting spacecraft using the sinusoidal autonomous trajectory in LEO for roll (yellow), pitch (pink), and yaw (blue).

Applying the sinusoidal trajectory equations generated previously, the simulation produced the following results which are displayed numerically in Table 4 and graphically in Figure 3, Figure 4 and Figure 5. The recorded parameters include the Euler angles, run time, and thrust required (which is proportional to the fuel consumption of the spacecraft), as well as the accuracy and precision of the simulation. It is to be noted that the accuracy of the simulation reached the maximum digit precision allowed by MatLab.

### 3.2. Pontryagin’s Autonomous Trajectory

**Figure 6:**
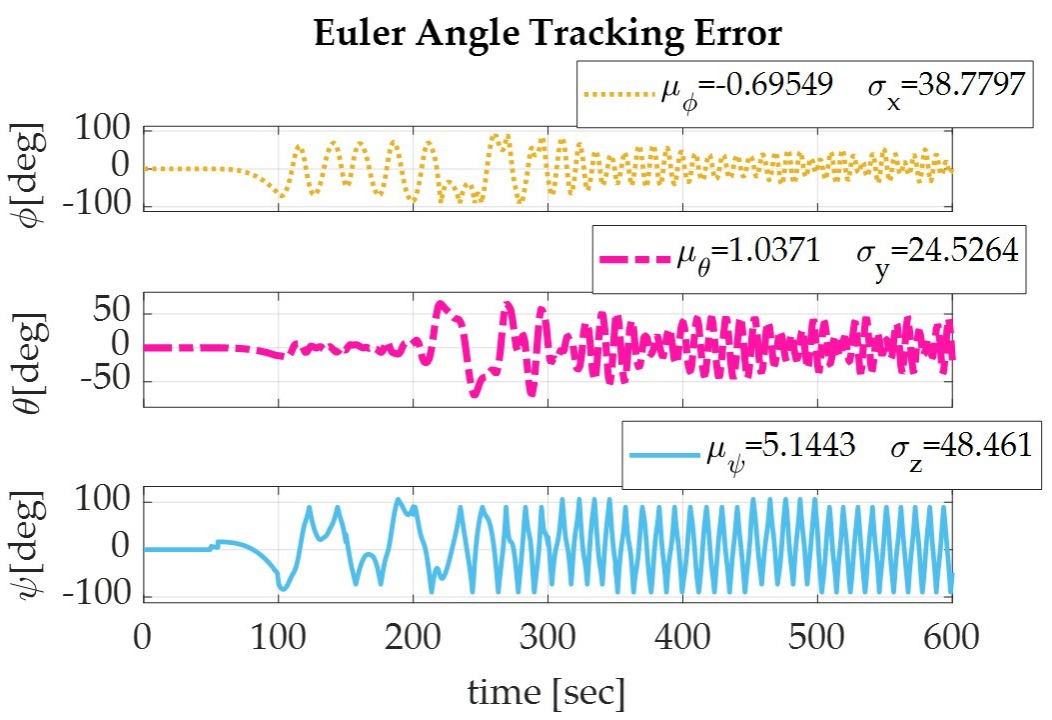
The Euler angle tracking errors for a spacecraft de-orbiting from LEO altitude (1000 km) using the Pontryagin’s autonomous trajectory for roll (yellow), pitch (pink), and yaw (blue).

**Figure 7:**
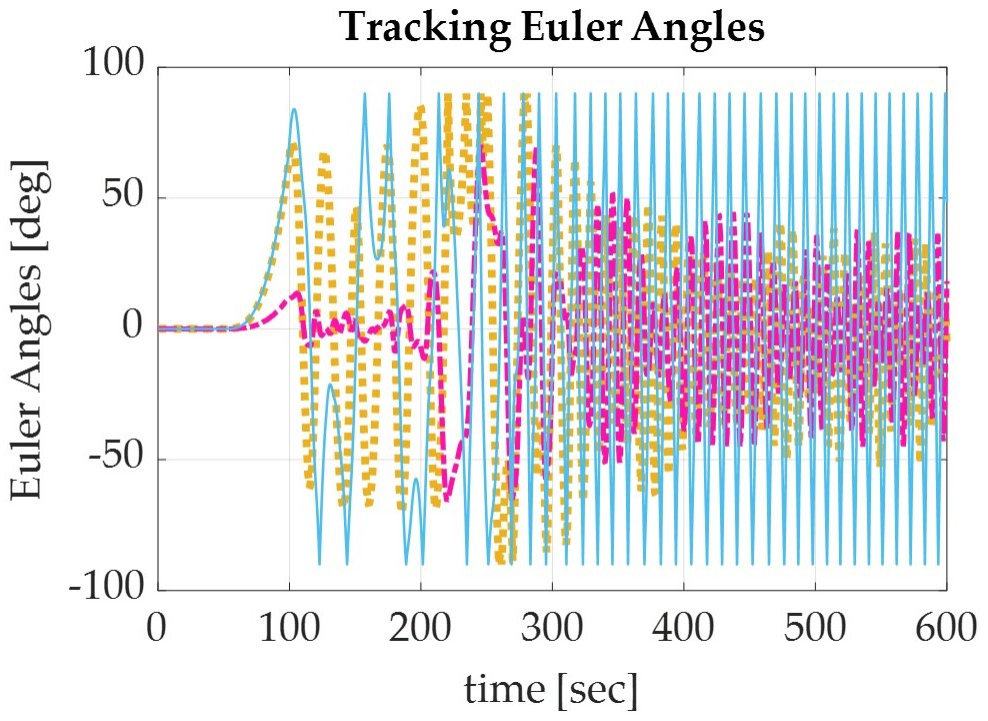
Graph of the Euler angle tracking for a spacecraft de-orbiting from LEO altitude (1000 km) using the Pontryagin’s autonomous trajectory for roll (yellow), pitch (pink), and yaw (blue).

**Figure 8:**
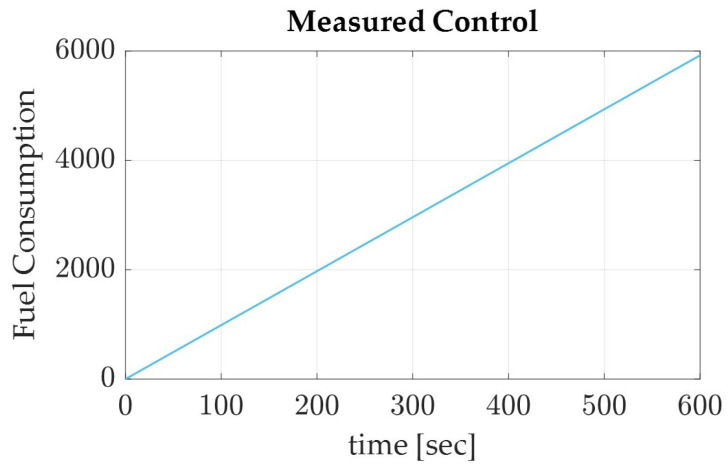
The control measured in terms of thrust applied, which can be estimated for fuel consumption, for a de-orbiting spacecraft using the Pontryagin’s autonomous trajectory in LEO.

In a similar effort, the generated equations for Pontryagin’s autonomous trajectory were applied to the simulation model. The results of the simulation are displayed numerically in Table 5 and graphically in Figure 6, Figure 7 and Figure 8. The recorded parameters include the Euler angles, total run time, and thrust required (which is proportional to the fuel consumption of the spacecraft), as well as the accuracy and precision of the simulation. It is to be noted that the accuracy of the simulation reached the maximum digit precision allowed by MatLab, equally precise as the sinusoidal trajectory results.

## 4. Discussion

With the results of the simulation completed using way-point guidance for both sinusoidal and Pontryagin’s autonomous trajectories, a discussion of how the results compare in relation to each other and with what was expected is presented in this section.

### 4.1. Results Validation

**Figure 9:**
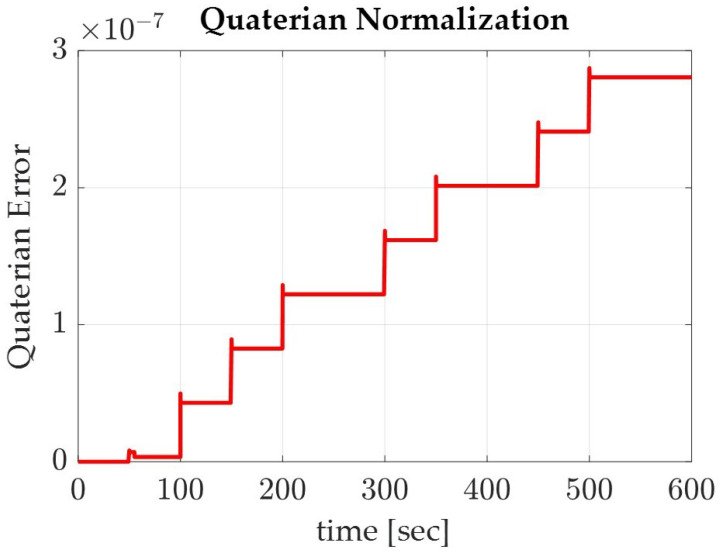
Graph of the normalized quaternions for the spacecraft on the sinusoidal trajectory.

**Figure 10:**
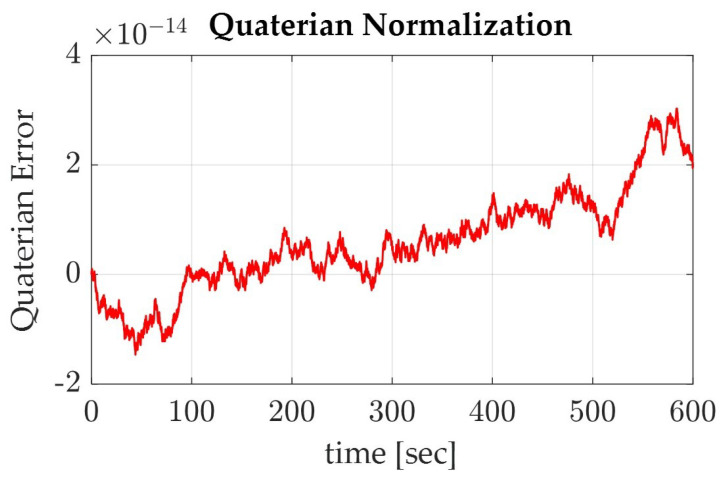
Graph of the normalized quaternions for the spacecraft on Pontryagin’s trajectory.

To verify the accuracy of the model, the quaternions were normalized and monitored. The quaternions should remain constant throughout the simulation, so plotting the normalization of the quaternions should graphically depict the accuracy of the simulation. As shown in Figure 9 and Figure 10, the differences within the normalized quaternions are very small, on the order of $10^{-7}$ for the sinusoidal trajectory and on the order of $10^{-14}$ for Pontryagin’s trajectory. It is also noted that the sinusoidal trajectory produced the larger, more step-like quaternion deviations when compared with that of the smaller, more jitter-like error in Pontryagin’s trajectory.

### 4.2. Comparison between the Sinusoidal and Pontryagin Trajectory Results

With the results verified in terms of the maximum digit precision available and monitored accuracy in regards to the normalized quaternions, a discussion of the results can be made. In comparing the results of the Euler angles, Pontryagin’s trajectory produced a lower yaw with higher roll and pitch degrees than the sinusoidal trajectory (Table 4 and Table 5). This could be attributed to the optimization of fuel consumption in relation to the slew maneuver, as the Pontryagin trajectory conserved over five magnitudes of fuel more than the sinusoidal trajectory, the traditional autonomous trajectory generation method. Pontryagin’s method has the potential to save 37.9% of fuel when used to de-orbit a spacecraft from LEO using collision avoidance techniques (Figure 5 and Figure 8). When comparing the computation time of Pontryagin’s autonomous trajectory generation to that of the sinusoidal approach, Pontryagin’s method was over 15 min faster and has the capability to save 40.5% more time than if the trajectory was generated with a sinusoidal approximation. Overall, the results of the simulation prove that Pontryagin’s method is more optimal for autonomous trajectory by conserving 37.9% more fuel and 40.5% more time than the sinusoidal autonomous trajectory.

### 4.3. Future Research

The results of the simulation proved to be fruitful, as the autonomous trajectory generated from Pontryagin’s method outperformed the sinusoidal trajectory, as expected. This opens the door for further integration of optimization techniques for a more artificially intelligent collision avoidance approach to spacecraft de-orbiting maneuvers. Additionally, another area for future research could be applied to increase the breadth of the simulation to include a touch down on the Earth’s surface. With this expansion of the current Simulink model, it could make the model more applicable to the current needs of the aerospace industry.

## Figures and Tables

**Table 1:** The 6 DoF Motion Equation Variable Definitions.

Variable	Definition	Variable	Definition
*F*	Applied force	*m*	Mass
*r*	Displacement Radius vector relative to rotating frame	*v*	Velocity Radius vector relative to rotating frame
*a*	Translational acceleration	dt	Time step
ω	Angular velocity	dω	Angular acceleration

**Table 2:** Sinusoidal Trajectory Variable Definitions.

Variable	Definition	Variable	Definition
$z_{d}$	Position motion state	*t*	Current time of the maneuver
$\dot{z_{d}}$	Velocity motion state	$t_{maneuver}$	User-defined duration of maneuver
$\ddot{z_{d}}$	Acceleration motion state	$t_{quiescant}$	User-defined quiescent period used to validate code
*A*	Motion state displacement amplitude	$A_{0}$	Motion state initial displacement amplitude

**Table 3:** Pontryagin’s Trajectory Variable Definitions.

Variable	Definition	Variable	Definition
$f,f^{*}$	Variable force and optimal force	$t_{0},t_{f}$	Used-Defined initial and final time
$v,\dot{v},v_{0},v_{1},v^{*}$	Velocity motion state, rate of velocity, initial and final velocity, and optimal velocity	[M]	Mass matrix
$x,\dot{x},x_{0},x_{1},x^{*}$	Position motion state, rate of position, initial and final position, and optimal position	*u*	Control variable and fuel consumption approximation

**Table 4:** Simulation results of the sinusoidal trajectory.

Parameter	Units	Value
Final Roll Position	degrees	$5.1687\times 10^{-8}$
Final Pitch Position	degrees	$9.9275\times 10^{-8}$
Final Yaw Position	degrees	−30.0000
Total Run Time	seconds	2257.4624
Total Thrust Required	N	$2.8587\times 10^{9}$
Precision	-	$2.2204\times 10^{-16}$

**Table 5:** Simulation results of the Pontryagin trajectory.

Parameter	Units	Value
Final Roll Position	degrees	−6.7792
Final Pitch Position	degrees	18.2819
Final Yaw Position	degrees	18.1840
Total Run Time	seconds	1343.5235
Total Thrust Required	N	$1.7765\times 10^{4}$
Precision	-	$2.2204\times 10^{-16}$

**Figure A1:**
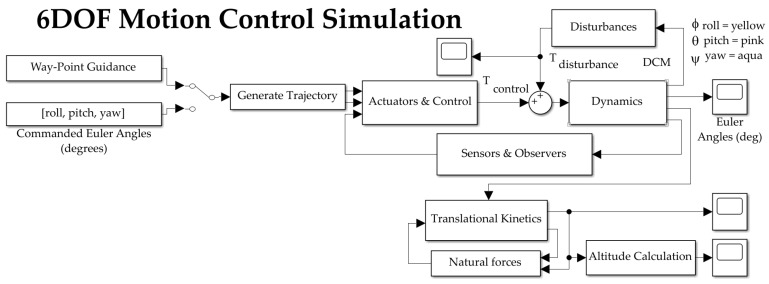
MatLab model with complete display of all Simulink subsystems.

**Figure A2:**
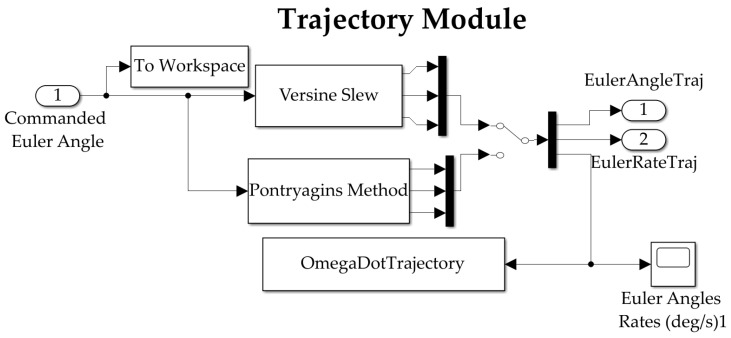
MatLab model with complete detailed display of the trajectory Simulink subsystem.

**Figure A3:**
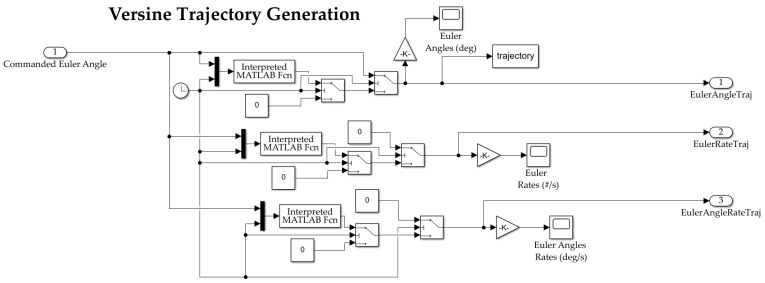
MatLab model with complete detailed display of the sinusodial generation block in the trajectory Simulink subsystem.

**Figure A4:**
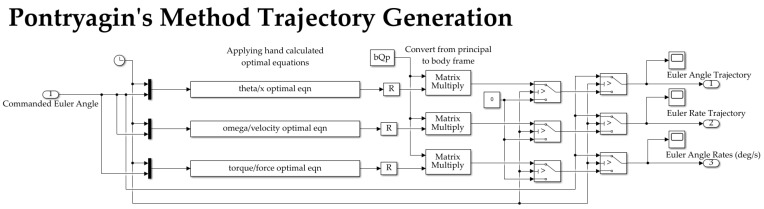
MatLab model with complete detailed display of optimal trajectory generation using Pontryagin’s method block in the trajectory Simulink subsystem.

**Figure A5:**
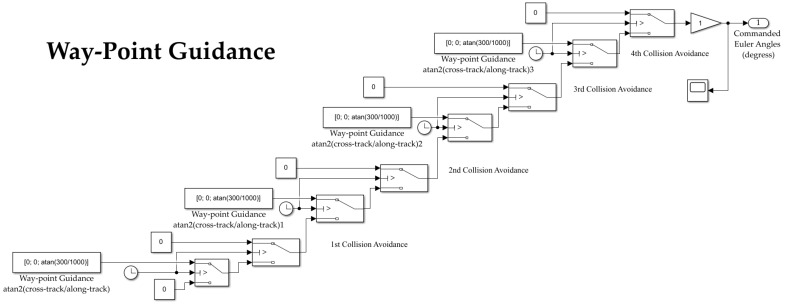
MatLab model with complete detailed display of the way-point guidance Simulink subsystem.

**Figure A6:**
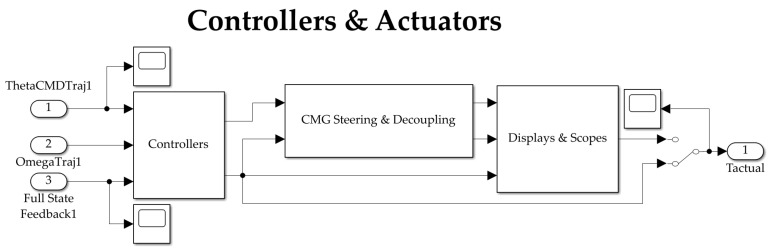
MatLab model with complete detailed display of the controllers and actuators Simulink subsystem.

**Figure A7:**
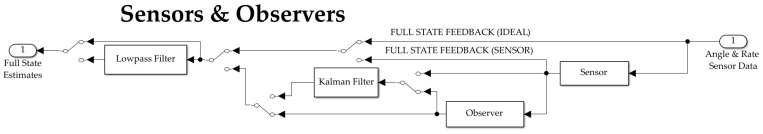
MatLab model with complete detailed Display of the sensors and observers Simulink subsystem.

**Figure A8:**
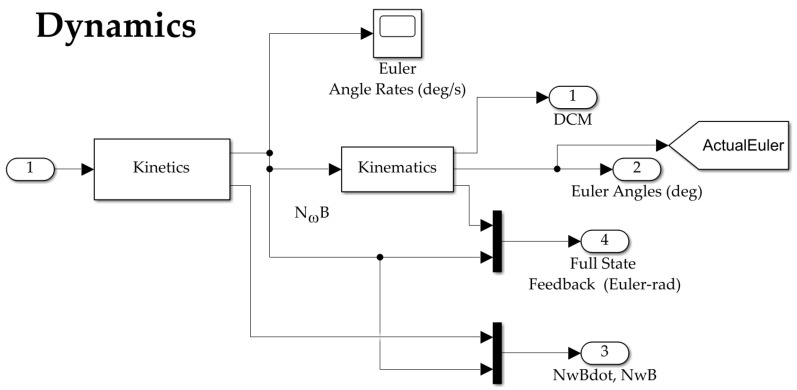
MatLab model with complete detailed display of the dynamics Simulink subsystem.

**Figure A9:**
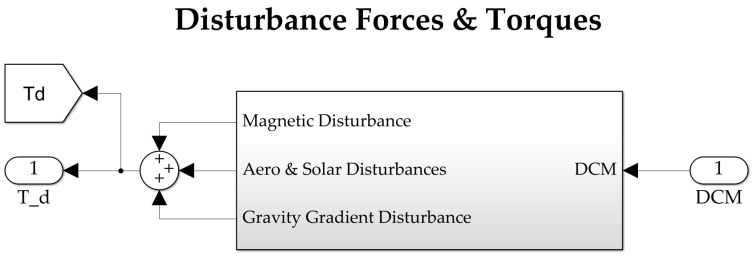
MatLab model with complete detailed display of the spacecraft disturbances Simulink subsystem.

**Figure A10:**
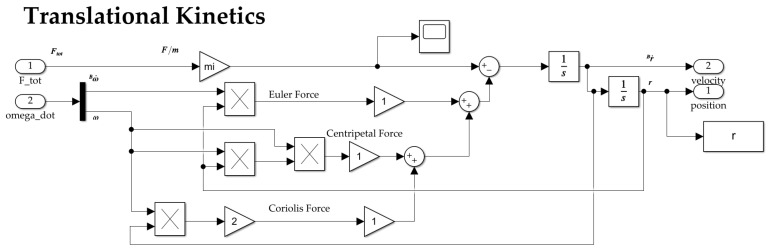
MatLab model with complete detailed display of the kranslational Kinetics Simulink subsystem.

**Figure A11:**
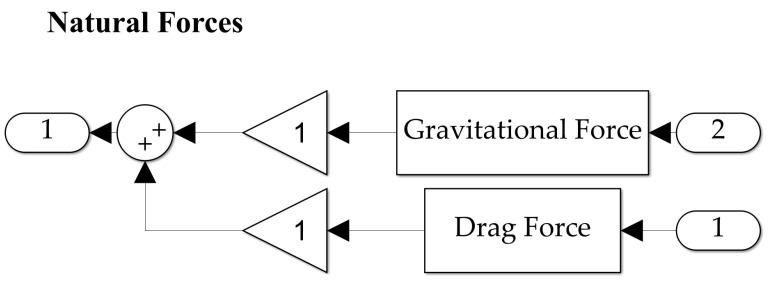
MatLab model with complete detailed display of the natural forces Simulink subsystem.

## Data Availability

Data may be made available by contacting the corresponding author.

## Abbreviations

The following abbreviations are used in this manuscript:

LEO	Low-Earth Orbit
NASA	National Aeronautics and Space Administration
NORAD	North American Aerospace Defense Command
